# Impact of Natural Disasters on Mental Health: A Cross-Sectional Study Based on the 2014 China Family Panel Survey

**DOI:** 10.3390/ijerph19052511

**Published:** 2022-02-22

**Authors:** Rui Zhang, Yunzhi Zhang, Zhe Dai

**Affiliations:** 1 Department of Economics, Jinan University, Guangzhou 510632, China; njuetimo@163.com; 2 Faculty of Law, Economic, and Management, LEO-University of Orléans, 45067 Orléans, France; 3 Law School, Jinan University, Guangzhou 510632, China; daizheking@126.com

**Keywords:** natural disasters, mental health, happiness, life satisfaction

## Abstract

Mental health problems are a leading cause of disability in both developed and developing countries, and the consequences of mental health disorders for individuals, families, and society as a whole could be severe and costly. To supplement relevant research and provide insightful policy suggestions to families, government and societies, this study investigates the nexus between natural disasters and mental health for middle-aged and older adults in rural China. Based on data of 8721 observations from the 2014 China Family Panel Studies, we estimate the effects of natural disasters on mental health using ordinary least squares and propensity score matching. Our findings suggest that natural disasters have a significant negative effect on middle-aged and older adults’ mental health in the case of rural China. This effect is heterogeneous depending on individuals’ education level and their agricultural production status. Finally, individuals’ happiness and life satisfaction are shown to be the potential mechanism through which the effect of natural disasters on mental health operates.

## 1. Introduction

Natural disasters, which are a possible result of global warming, play a crucial role in the relationship between humans and nature. For this reason, natural disasters have been widely studied, with researchers exploring their impact on society and aspects such as household finance, poverty, family violence, the macroeconomy, and energy consumption [1,2,3,4,5,6]. It is obvious that natural disasters threaten people’s lives and physical health; however, little attention has been paid to their impact on mental health. Natural disasters can cause anxiety, sleep disturbances, impaired interpersonal relationships, and depression, among other mental problems [7,8,9].

The importance of mental health is confirmed by the World Health Organization [10], which states that: “Health is a state of complete physical, mental and social well-being and not merely the absence of disease or infirmity”. Mental health is closely related to daily life and work, and it affects our attitude to life and work productivity. Research also indicates that mental illness can be costly for individuals and society [11,12,13,14,15,16]. Serious mental illness can even lead to suicide. Globally, around 703,000 people die by suicide every year (World Health Organization, 2021) [10]. Hence, exploring the causes of mental health problems is crucial for the well-being of individuals, their families, and society as a whole.

Given the importance of mental health, the determinants of mental health problems attract scholars’ interests. Most of the research focuses on the impact of human activity on mental health. For example, Ettner [17] found that an increase in individuals’ income can improve their mental health. In contrast, unemployment has a negative impact on mental health, a relationship that has been investigated by Scutella and Wooden [18]. Furthermore, Chen and Fang [19] reveal that China’s one-child policy has a negative impact on elderly people’s mental health. Apart from the abovementioned aspects, external shocks—such as economic shocks and war—can also affect mental health [20,21,22]. Recent trends have led to a proliferation of studies about the link between environment and health.

Environmental problems represent one of the most pressing concerns for global health in the 21st century. Specifically, it has been shown that air pollution can exacerbate respiratory or heart disease, among others [23,24,25,26]. Employing two-stage least squares estimation with data from the China Migrant Dynamic Survey, Gu et al. [27] found that poor air quality could cause tension, depression, and irritability, which could further harm mental health. Compared with air pollution, natural disasters are difficult to control and deal with for human beings. Some studies investigate the possible impact of specific natural disasters on mental health. For example, Yokoyama et al. [28] found that earthquakes and tsunamis have a negative impact on disaster survivors. The study of Gissurardóttir et al. [29] indicates that exposure to a volcanic eruption may cause mental health disorders. After the outbreak of COVID-19, most analyses focused on the impact of the pandemic on mental health. Pfefferbaum and North [30] found that the COVID-19 pandemic may result in a negative impact on individuals’ mental health. This result was also confirmed by Zhang and Ma [31], Yao et al. [32] and Li et al. [33]. Furthermore, many studies explore temperature as a factor that can impact mental health [16,34,35,36] or even lead to suicide [37]. Although the existing literature has investigated the determinants of mental health from different perspectives, little attention has been paid to the essential role of natural disasters affecting humans’ mental health in rural China.

This paper seeks to fill a gap in the related literature and to understand the relationship between natural disasters and human beings. We employ data from the China Family Panel Studies (CFPS) [38] in 2014 to identify the impact of natural disasters on mental health for middle-aged and older adults in rural China. We focus on rural China for three reasons. First, given the vulnerability of the infrastructure in China, natural disasters have a more devasting potential to affect this country. Over the years, economic development has been China’s main goal, with individual interests being subordinated to the collective interest. The huge population base detracts from the value of the individual, not to mention the importance of their health. Second, natural disasters have a longer and more persistent destructive impact on rural regions’ infrastructure than in urban regions. Third, natural disasters directly influence farmers’ daily life and work. Moreover, middle-aged, and older people constitute the main source of labor for most families in China. Another reason that we focus on this cohort is that China is rapidly aging. The seventh Population Census (2021) [39] shows that the share of people over 60 in the total population is 18.7%, accounting for 264 million people. This number has increased by 5.44%, compared to 2010. The mental health problems of middle-aged and older adults can affect the quality of development in China. 

The novelty of this paper is four-fold. First, to the best of our knowledge, this is the first paper to investigate the impact of natural disasters on mental health for the case of a developing country: China. Previous studies focused on the impact of a specific disaster, such as heat, floods, hurricanes, and earthquakes on mental health [9,40,41,42]. Our research investigates the general impact of natural disasters as an external shock on middle-aged and older adults’ mental health. Secondly, we address the impact on a particular cohort; specifically, middle-aged and older people in a rural region, which is important to discuss regarding this issue. Third, we examine the heterogeneity of effects by splitting the sample into different education levels and agricultural production status. These results help us to understand the heterogeneity of the impact of natural disasters on mental health. Finally, existing studies fail to provide mechanisms as to how the natural disaster could impact mental health [43,44]. This paper reveals that natural disasters could affect mental health through the influence of happiness and life satisfaction. 

The remainder of this paper is structured as follows. In Section 2, we describe the study design, statistical analysis, data, and methodology. The empirical analysis is reported in Section 3. In Section 4 we discuss the results and provide the policy implications. Conclusions are drawn in Section 5.

## 2. Materials and Methods

### 2.1. Study Design

A cross-sectional study was performed by using the 2014 China Family Panel Studies (CFPS) [38], a nationwide, comprehensive, longitudinal survey in mainland China. Five follow-up sampling waves were conducted in 2010, 2012, 2014, 2016, and 2018. However, only the 2014 CFPS has complete information on natural disasters and mental health. Thus, only the 2014 baseline survey is used for the analysis in this study. From July 2014 to May 2015, the CFPS project team collected data at individual, family, and community levels through face-to-face interviews and telephone surveys. CFPS sampling adopts implicit stratified, multi-stage, multi-level, and proportional probability sampling. The administrative division and socio-economic level are the main hierarchical variables. The samples of each sub-sample box of CFPS are extracted through three stages. The first stage sample is the administrative district/county. The second stage sample is administrative village/neighborhood committee, and the third stage (terminal) sample is household. Twenty-five provinces or their administrative equivalents were surveyed: Beijing, Chongqing, Shanghai, Tianjin, Zhejiang, Liaoning, Fujian, Sichuan, Shandong, Guizhou, Gansu, Hebei, Hubei, Hunan, Guangdong, Guangxi, Yunnan, Heilongjiang, Jilin, Shanxi, Anhui, Jiangxi, Shaanxi, Henan, and Jiangsu. The data included individual, family, and community levels; that is, individual psychological and physiological status, education outcomes, natural disaster, demographic characteristics, and family economic characteristics. 

In 2014, the number of middle-aged and older adults (“middle-aged and older adults” refers to individuals older than 44 years old) in the sample was 18,607. Exclusion of outliers, urban, and missing data yields 8721 observations. An econometric analysis using ordinary least squares (OLS) was conducted to investigate the effect of natural disasters on middle-aged and older adults’ mental health. This study was approved by the Ethics Committee of the Institute of Social Science Survey of Peking University, and ethical clearance or equivalent approval to conduct the study was granted in each country.

Our paper not only investigated the impact of natural disasters on middle-aged and older adults’ mental health, but also considered the heterogeneous effects and mechanisms of natural disasters on mental health. Hence, five hypotheses were proposed for our study.

Natural disasters may influence individuals’ life through different aspects. For example, natural disasters may damage individuals’ houses and crops, resulting in huge financial stress for disaster survivors. Furthermore, anxiety, impaired interpersonal relationships, food insecurity, and numerous other potential triggers for stress response may all have been intensified due to natural disasters [7,8,9]. Based on the above analysis, we propose Hypothesis 1.

**Hypothesis** **1.** *Natural disasters have a significant negative effect on middle-aged and older adults’ mental health.*

Belo et al. [44] found that well-educated people tend to have a higher income, a healthy diet, and an optimistic attitude towards life. Natural disasters might destroy immovables, cause massive loss of human life, and destruction of resources. Compared with less-educated individuals, well-educated people own more social and economic resources. Those well-educated individuals could better cope with the negative impact of natural disasters. The effect of natural disasters on mental health might not be homogeneous for people at different education levels. Hence, Hypothesis 2 arises.

**Hypothesis** **2.** *The impact of natural disasters for well-educated individuals is less strong than it is for their less-educated counterparts.*

Most individuals have sustained heavy financial losses due to natural disasters. People involved in agricultural production suffer more losses from natural disasters [45]. Furthermore, property loss induces anxiety or other mental health problems in these people. Second, compared with the individuals who are not involved in agricultural production, natural disasters can be more devastating for those who are. Since the damage affects not only financial property, but also people’s agricultural livelihoods [3], the double loss might result in mental health problems. The effect of natural disasters on mental health might also vary depending on the family’s agricultural production status. Hence, we propose Hypothesis 3.

**Hypothesis** **3.** *Individuals show a stronger response to natural disasters if they have a family member involved in agricultural production, compared to those who do not.*

Individuals with a higher level of happiness have more positive emotions and attitude to life than the ones with a lower level. Previous studies have recognized the important role of happiness in an individual’s mental health [46]. Furthermore, the existing literature indicates that natural disasters have a significant negative impact on individuals’ happiness [47,48]. Hence, we propose Hypothesis 4.

**Hypothesis** **4.** *Natural disasters have an impact on mental health through their effects on happiness.*

Natural disasters are linked with reduced satisfaction. Effects of natural disasters on life satisfaction fall into two broad categories: psychic costs and financial losses. Luechinger and Raschky [49] found that flood disasters have a negative effect on individuals’ life satisfaction. Individuals’ life satisfaction scores embody specific information on a subjective assessment of their daily life. Respondents with a higher score of life satisfaction are less likely to experience a psychological problem. Hence, Hypothesis 5 is proposed.

**Hypothesis** **5.** *Natural disasters can harm mental health through their effects on life satisfaction.*

### 2.2. Statistical Analysis

Statistical analysis was conducted using econometric software STATA version 15.1 (StataCorp, College Station, TX, USA). We report the mean, standard deviation, minimum, and maximum of variables in Table 1. Given mental health is a continuous variable, OLS was constructed to investigate the causal relationship between natural disasters and middle-aged and older adults’ mental health. (We used the STATA package “regress” for the OLS regression). In our robustness check, we estimate the effect of natural disasters on mental health using propensity score matching (PSM). (We used the STATA package “psmatch2” to calculate the average treatment effect on the treated (ATT) of the various propensity score matching methods). To investigate the mechanisms, we estimate the impact of natural disasters on individuals’ happiness and life satisfaction using OLS and the ordered probit model. (We used the STATA package “oprobit” for the ordered probit model). All reported *p*-values were two-tail. The level of statistical significance was set at p<0.1. 

### 2.3. Variables and Descriptive Statistics

Outcome variable: middle-aged and older adults’ mental health

The main outcome variable in this paper is the mental health of middle-aged and older adults in rural China. Following existing studies [43,50], the mental health index is derived from the 6-item short form of the Center for Epidemiologic Studies of Depression (CES-D) in the CFPS. (CES-D questions: 1. How often did you feel depressed that nothing could cheer you up during the past 30 days? 2. How often did you feel nervous during the past days? 3. How often did you feel restless or fidgety during the past 30 days? 4. How often did you feel hopeless during the past 30 days? 5. How often did you feel that everything was an effort during the past 30 days? 6. How often did you feel that life was meaningless during the past 30 days? Individuals were asked to indicate the frequency of their feelings on a five-scale metric—“Almost daily”, “Often”, “Half of the time”, “Sometimes”, and “Never”. These responses are coded from 1 to 5, respectively). The response for each question is coded from 1 to 5. There are six questions to assess mental state in the survey, and each one is constructed and standardized to have a mean of zero and a standard deviation of one. The final score is calculated by aggregating the multiple measures into indices. The higher the index value, the better the individual’s mental health.

Independent variable: natural disaster

We consider two measures of natural disaster as the independent variable. The first one is captured by a dummy variable (Disaster_d). It equals 1 if the middle-aged or older adult has experienced at least one type of natural disaster, and otherwise 0. (The types of natural disasters include typhoons, floods, storm surges, forest fires, frost, hail, landslides, debris flow, earthquakes, infectious diseases, agricultural and forestry pests, etc.). The second is constructed as a continuous variable (Disaster_n), which measures the number of types of natural disaster that the middle-aged or older adult has experienced.

Control variables and descriptive statistics

We include the following control variables: age, a dummy variable for sex, education level, marital status, cognitive abilities, income, medical insurance, and a dummy variable for agricultural production. In addition, we control for family size, house value, and family expenditure. Descriptive statistics of the variables used in the paper are reported in Table 1, where it can be seen that the sampled middle-aged and older adults were 58.41 years old on average, and 50.4 percent of them were male. The average mental health score is −0.339. About 75 percent of middle-aged and older adults have experienced at least one type of natural disaster. The value of Disaster_n varies from 0 to 5. That is to say, the most types of disasters that have been experienced by a person is 5, and the least is 0 in our sample. 

### 2.4. Empirical Methodologies

The effect of natural disasters on middle-aged and older adults’ mental health is estimated using ordinary least squares, as follows:

(1) $$mental_{i}=\alpha_{0}+\beta_{0}disaster\_d+\lambda control_{i}+\varepsilon_{i}$$

(2) $$mental_{i}=\alpha_{0}+\beta_{0}disaster\_n+\lambda control_{i}+\varepsilon_{i}$$

where $mental_{i}$ represents the dependent variable (middle-aged and older adults’ mental health), disaster_d represents the natural disaster dummy variable (dummy variable equal to 1 if the middle-aged or older adult experienced at least one type of natural disaster, and otherwise 0), disaster_n represents the number of times a natural disaster was experienced, and $control_{i}$ is a vector of observable determinants of middle-aged and older adults’ mental health.

## 3. Empirical Results

### 3.1. The Basic Correlation

**Figure 1:**
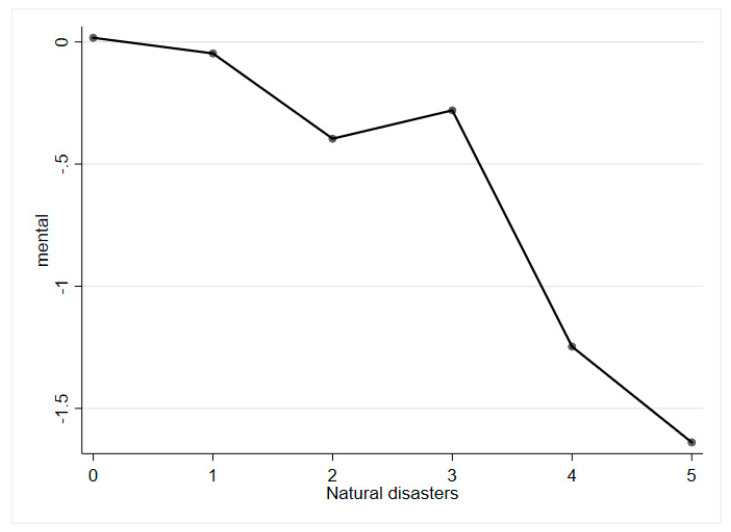
Graph of natural disasters and mental health.

The basic relationship between natural disasters and mental health is presented in Figure 1. The graph indicates that a negative correlation exists between natural disasters and middle-aged and older adults’ mental health.

### 3.2. Baseline Results

When investigating the causal relationship between natural disasters and mental health, an individual’s math and language abilities are generally highly correlated. High correlation among variables gives rise to concerns about multicollinearity, which may lead to considerable bias in the estimation. We use the variable inflation factor (VIF) to check for multicollinearity in our model. Table 2 reports the VIF of each variable. In each case, the VIF is less than the rule-of-thumb value of 10, indicating that multicollinearity is not a major issue.

Table 3 reports the baseline results on the effects of natural disasters on middle-aged and older adults’ mental health. Columns (1) and (3) include only the dummy of natural disasters and the intensity of natural disasters, respectively. A set of control variables affecting middle-aged and older adults’ mental health is included in columns (2) and (4). The effects in columns (1) and (3) suggest a salient negative effect of natural disasters on mental health for middle-aged and older people. When controlling for a set of covariates in columns (2) and (4), results from OLS models indicate that natural disasters are a significant predictor of middle-aged and older adults’ mental health, showing a negative correlation. Those results verify Hypothesis 1. In addition, sex shows a positive sign in columns (1) and (3). This indicates that males have better mental health than females. The results for education report positive signs, indicating that education has a positive impact on mental health. The coefficients of marital status are positive and statistically significant. The results indicate that the mental health status of married adults is higher than in their unmarried counterparts. Math abilities, income, insurance, and house value show a salient positive impact on mental health.

### 3.3. Endogeneity

Bearing selection bias in mind, we estimate the causal effect of natural disasters on mental health using the propensity score matching (PSM) technique. In this case, we use a dummy variable equal to 1 if the middle-aged or older adult experienced at least one type of the natural disaster (treatment group), or otherwise 0 (control group).

**Figure 2:**
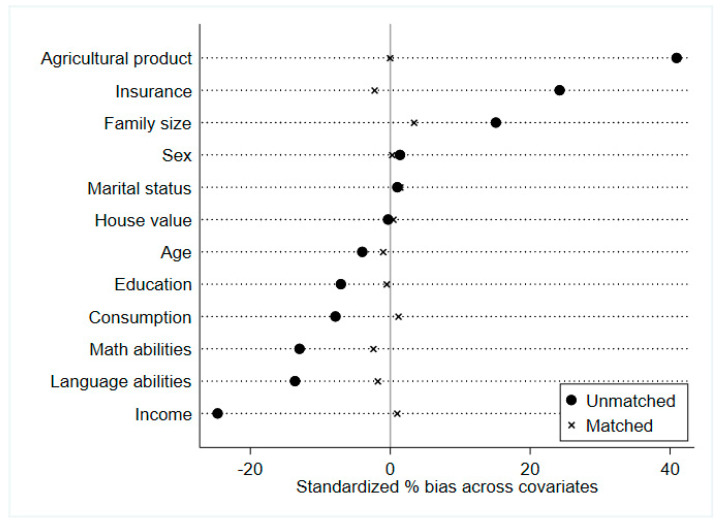
Standardized bias before and after matching.

An important step when applying PSM is to check the covariate balance of the treatment and control group, which is achieved if both groups have similar observable covariates. This paper uses two methods to check the covariate balance of the two groups. The first one is essentially based on comparing the mean (after matching) of observable covariates in the two groups. The second one is based on the standardized bias. Table 4 reports the results of the mean of the observable covariates in the two groups. The results in column (5) indicate that the *p*-values (after matching) are larger than 0.1 in most of the cases. Additionally, we report the standardized bias in Figure 2. The standardized bias reduction is below 5%, providing evidence that the covariates are balanced in the two groups.

**Figure 3:**
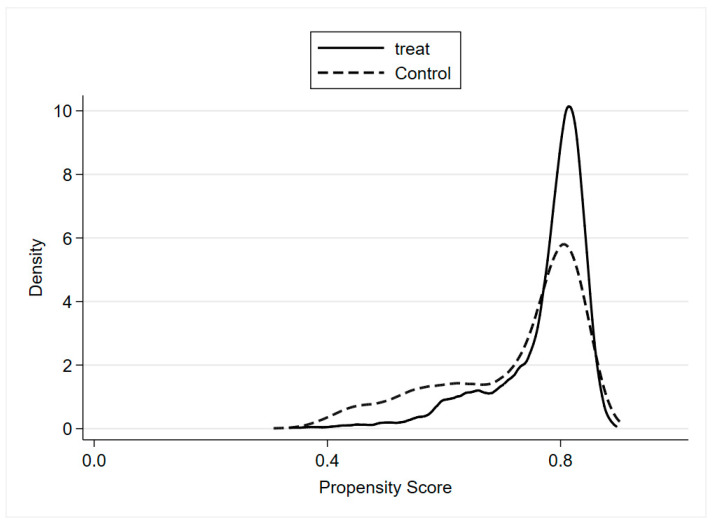
Density distribution of the propensity score (before matching).

**Figure 4:**
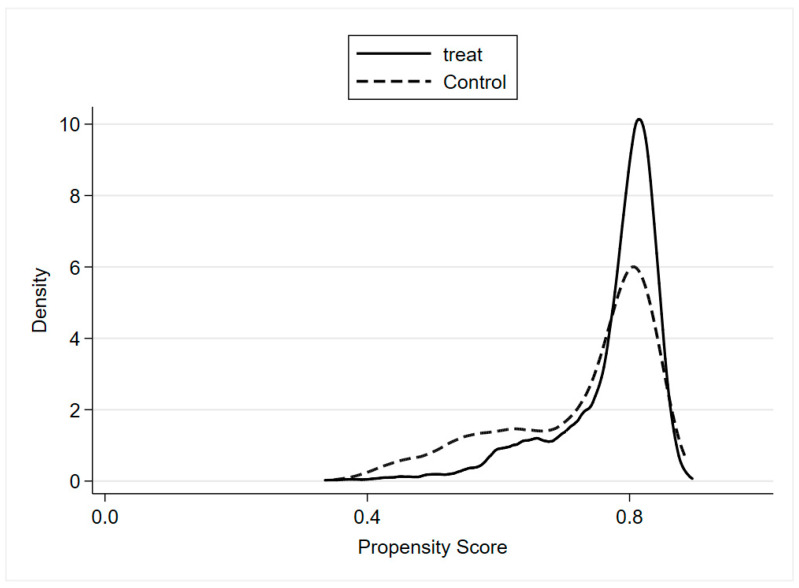
Density distribution of the propensity score (after matching).

According to Heckman et al. [51], a crucial step when applying PSM is to examine the overlap and region of common support between treatment and control groups. Figure 3 and Figure 4 report the estimation of the density distribution in the two groups, indicating that most samples fall into the region of common support.

Following Rosenbaum and Rubin [52], this paper presents different types of matching estimators, including kernel matching, local linear matching, radius matching, and nearest-neighbor matching (k = 1, k = 4). Table 5 presents the results of the different matching strategies. The results indicate that the average treatment effect on the treatment of the various matching methods is all negative and significant, indicating a negative correlation between natural disaster and mental health in our sample. 

### 3.4. Robustness Check

Two lines of the robustness check are conducted to confirm the reliability of the results in the previous section. In the first, we use an alternative measure of mental health. In the second, we employ another methodology to estimate the effect of natural disasters on middle-aged and older adults’ mental health.

One concern may be that our findings might be driven by the measurement of mental health. To analyze this, we construct an alternative index to measure mental health in our sample. We adopt a factor analysis approach to assess the individual’s mental health. The results show that the KMO-statistics are all larger than 0.8, Cronbach’s alpha is 0.86, and the *p*-values of the Bartlett test of sphericity are all less than 0.01, confirming that exploratory factor analysis fits well as a method to measure mental health. We also use this index to regress our model using OLS, and the results are reported in Table 6. The results indicate that natural disasters have a negative effect on middle-aged and older adults’ mental health, consistent with the OLS results of Table 3.

In the second robustness check, we apply the probit methodology to estimate the effect of natural disasters on middle-aged and older adults’ mental health. To do this, we replace individuals’ mental health by using a dummy. This dummy takes 1 if the value of mental health is more than −0.339, and otherwise 0. Columns (3)–(4) of Table 6 show the estimated results, which indicate that natural disasters have a negative effect on mental health. We also report the margin effects in columns (5)–(6) of Table 6. The marginal effects are all negative and statistically significant. All in all, the results in Table 6 are consistent with the results in Table 3.

### 3.5. Heterogeneity

To better understand the relationship between natural disasters and mental health, we examine the heterogeneity of effects by splitting the sample into different education levels and agricultural production status. 

In order to check Hypothesis 2, Table 7 presents the results of the heterogeneous effect of natural disasters on mental health for different education levels. The results suggest that less-educated adults show a stronger response to natural disasters than well-educated ones. These results verify Hypothesis 2.

To check Hypothesis 3, we include a dummy variable to measure the agricultural production status in a family. The dummy equals 1 if the individual belongs to a family involved in agricultural production, and otherwise 0. Table 8 reports the effect of natural disasters considering the family’s agricultural production status. The results indicate that middle-aged and older adults have a stronger response to natural disasters if they have a family member involved in agricultural production, compared to those that do not. The results in Table 8 verify Hypothesis 3. 

### 3.6. Mechanisms

To explore the mechanisms through which natural disasters affect middle-aged and older adults’ mental health, two channels are studied in this section: happiness and life satisfaction.

To test Hypothesis 4, we estimate the impact of natural disasters on happiness for middle-aged and older adults by means of OLS. (The happiness index ranges from 1–10, where 0 is the least happy and 10 is the most happy). Given that happiness is reported on an ordinal scale, we also employ the ordered probit model to investigate the impact of natural disasters on mental health. Table 9 reports the results for the effect of natural disasters on middle-aged and older adults’ happiness, revealing negative and statistically significant coefficients. This indicates that natural disasters have an impact on middle-aged and older adults’ mental health through their effects on happiness. The results in Table 9 verify Hypothesis 4.

In order to test Hypothesis 5, we investigate whether natural disasters can affect life satisfaction. This indicator is also available in the CFPS survey, with a higher value meaning higher life satisfaction. (The life satisfaction index ranges from 1–5). The corresponding estimates are presented in Table 10. Columns (1)–(2) of Table 10 report the results of the OLS. Life satisfaction is reported on an ordinal scale, which allows us to estimate the effects of natural disasters on mental health with the ordered probit model. The results show that the coefficients of the natural disasters are negative and statistically significant, indicating that natural disasters can harm mental health through their effects on life satisfaction. These results verify Hypothesis 5.

## 4. Discussion

There are a number of studies that are related to what we have examined in this paper, but they are in the spirit of focusing on a specific disaster. For instance, Kovats and Hajat [40] conducted a meta-analysis of previous studies and found that extreme hot weather threatens public health and can also be a cause of mortality. Furthermore, studies by Rataj et al. [41] and Weilnhammer et al. [53] show that extreme weather has a negative impact not only on physical health, but also on mental health. However, the abovementioned research is based on descriptive studies and lacks empirical support. Our research employs ordinary least squares and propensity score matching to investigate the causal impact of natural disasters on middle-aged and older adults’ mental health and provides empirical evidence on the effects of natural disasters on mental health. The baseline results are in line with previous studies [54,55]. Moreover, most of the research is derived from studies of flood-exposed regions. Unlike the research of Fernandez et al. [56], our research is derived from large-scale micro population survey data (CFPS). Based on Adult Psychiatric Morbidity Survey data in England, Graham et al. [42] investigated the impact of storms and floods on individuals’ mental health, but their paper makes no attempt to provide the mechanism analysis. Our research not only investigates the impact of natural disasters on individuals’ mental health, but also strives to ascertain the mechanism between natural disasters and mental health. Furthermore, previous studies found that experiencing an earthquake may influence sleep quality and interpersonal relationships, or even lead to suicide [7,8].

Despite mounting evidence indicating that heat, floods, and hurricanes might cause a negative impact on individuals, little has been said to discuss the impact of all kinds of natural disasters as an external shock on middle-aged and older adults’ mental health. Furthermore, our research also indicates that the impact of natural disasters on middle-aged and older adults’ mental health is heterogeneous depending on individuals’ education level and their agricultural production status. Our study found that well-educated individuals have a weaker response to natural disasters than their less-educated counterparts. Middle-aged and older adults show a stronger response to natural disasters if they have a family member involved in agricultural production, compared to those that do not. Last but not least, our findings provide new evidence on the causal mechanism between natural disasters and middle-aged and older adults’ mental health.

However, this paper is limited in some facets. First, we estimate the short-run effects of natural disasters on middle-aged and older adults’ mental health. Regretfully, due to data constraints, we fail to consider the long-term effects of natural disasters. Second, as well as the data limitations, we measure mental health in a very general way. For instance, post-traumatic stress disorder (PTSD) is highly related to disaster survivors [29,55]. Given the lack of relevant data to PTSD, we do not investigate the impact of natural disasters on PTSD. Third, although we try our best to include the factors that might affect individuals’ mental health, the model could not include some further external factors that affect individuals’ mental health, which are difficult to measure. An interesting future research avenue could be projected on the long-term and dynamic effects of natural disasters on middle-aged and older adults’ mental health. Furthermore, research could also investigate the causal effect between natural disasters and a specific mental problem, such as PTSD.

Several policy implications can be derived from this analysis. First, our study suggests that natural disasters have a notable adverse impact on middle-aged and older adults’ mental health. Thus, the government and society as a whole might need to provide aid to the middle-aged and older adults who have suffered from natural disasters. This help should be targeted not only at infrastructure reconstruction and financial subsidies but also at effective mental health care. Particular attention should be paid to those people who have a low level of education and are involved in agricultural production. Finally, the government might also focus more on helping the middle-aged and older adults of disaster-stricken regions by improving their happiness and life satisfaction. 

## 5. Conclusions

Given the importance of mental health in daily life, there has been a growing amount of research on this topic. In this paper, we investigate the causal relationship between natural disasters and mental health in the case of middle-aged and older adults in rural China by using 8721 observations from 2014 CFPS survey data. One of the most important findings to emerge from this paper is that natural disasters have a negative impact on mental health.

Further analysis on heterogeneous effects is conducted by splitting the sample according to educational level and family agricultural production status. On the one hand, the results show that natural disasters have a slightly stronger impact on less-educated people than their better-educated counterparts. On the other hand, compared with those whose family members are not involved in agricultural production, those who are involved in agriculture show a stronger response to natural disasters. Our study also investigates the mechanisms through which natural disasters can have an impact on mental health, indicating that they influence mental health through their effect on the individual’s level of happiness and life satisfaction.

## Figures and Tables

**Table 1:** Descriptive statistics of the key variables.

Variable	Definition	Mean	SD	Min	Max
Mental health	Middle-aged and older adult mental health	−0.339	4.977	−22.98	3.788
Disaster_d	Dummy variable equals 1 if the individual experienced at least one type of natural disaster, and otherwise 0	0.759	0.428	0	1
Disaster_n	The total types of disasters	1.746	1.482	0	5
Sex	1 for male, 0 for female	0.504	0.500	0	1
Age	Individual’s age	58.41	9.463	45	85
Education	Years of education	4.793	4.223	0	16
Marital status	Dummy variable equals 1 if the individual is married, and otherwise 0	0.876	0.329	0	1
Math abilities	Cognitive abilities	4.639	4.402	0	24
Language abilities	Cognitive abilities	9.744	10.16	0	34
Income	Individual’s income (in log)	2.986	3.579	0	12.39
Insurance	Individual has social insurance (1 for yes)	0.910	0.287	0	1
Agricultural production	Dummy variable equals 1 if the individual’s family is involved in agricultural production, and otherwise 0	0.828	0.377	0	1
Family size	The number of people in the family	4.284	2.041	1	17
House value	House value (in log)	10.88	2.595	0	16.12
Consumption	Annual household expenditure (in log)	10.26	0.940	5.481	15.45
Happiness	Middle-aged and older adult happiness	7.261	2.341	0	10
Life satisfaction	Middle-aged and older adult life satisfaction	3.829	1.044	1	5

**Table 2:** The variance inflation factor of each variable.

Variable	VIF	VIF
Language abilities	2.560	2.560
Math abilities	2.390	2.390
Education	1.820	1.820
Age	1.370	1.370
Consumption	1.310	1.310
Family size	1.230	1.230
Sex	1.200	1.200
Income	1.170	1.170
Agricultural production	1.140	1.140
Marital status	1.130	1.130
House value	1.110	1.110
Insurance	1.060	1.060
Disaster_d	1.050	
Disaster_*p*		1.040
Mean VIF	1.430	1.420

Note: VIF represents variable inflation factor.

**Table 3:** OLS results of the effects of natural disasters on middle-aged and older adults’ mental health.

Variable	Dependent Variable: Mental Health			
	(1)	(2)	(3)	(4)
	OLS	OLS	OLS	OLS
Disaster_d	−0.470 ***	−0.358 ***		
	(0.124)	(0.124)		
Disaster_n			−0.290 ***	−0.267 ***
			(0.036)	(0.036)
Sex		0.639 ***		0.675 ***
		(0.115)		(0.114)
Age		−0.008		−0.008
		(0.007)		(0.006)
Education		0.079 ***		0.075 ***
		(0.016)		(0.017)
Marital status		1.018 ***		0.986 ***
		(0.193)		(0.168)
Math abilities		0.054 ***		0.058 ***
		(0.017)		(0.018)
Language abilities		0.006		0.004
		(0.008)		(0.008)
Income		0.041 ***		0.035 **
		(0.015)		(0.016)
Insurance		0.321 *		0.344 *
		(0.191)		(0.187)
Agricultural production		−0.291 *		−0.223
		(0.150)		(0.147)
Family size		0.006		0.012
		(0.028)		(0.028)
House value		0.154 ***		0.156 ***
		(0.023)		(0.021)
Consumption		−0.005		0.001
		(0.067)		(0.063)
Constant	0.017	−3.337 ***	0.167 **	−3.254 ***
	(0.108)	(0.910)	(0.082)	(0.846)
Observations	8721	8721	8721	8721
Adjusted R2	0.002	0.042	0.007	0.047

Note: *** *p* < 0.01, ** *p* < 0.05, * *p* < 0.10. Standard errors clustered at the individual level are reported in parentheses. OLS represents ordinary least squares.

**Table 4:** The mean of covariates in treatment and control groups.

Variable	Matching Status	Mean		T-Value	*p*-Value
		Treatment	Control		
Sex	Before	0.505	0.498	0.56	0.579
	After	0.504	0.502	0.13	0.893
Age	Before	58.322	58.704	−1.61	0.107
	After	58.329	58.429	−0.61	0.542
Education	Before	4.721	5.021	−2.84	0.005
	After	4.706	4.729	−0.31	0.754
Marital status	Before	0.877	0.874	0.40	0.687
	After	0.877	0.872	0.80	0.422
Math abilities	Before	4.501	5.075	−5.22	0.000
	After	4.488	4.596	−1.43	0.154
Language abilities	Before	9.410	10.797	−5.46	0.000
	After	9.392	9.576	−1.05	0.295
Income	Before	2.768	3.673	−10.15	0.000
	After	2.760	2.725	0.58	0.559
Insurance	Before	0.938	0.852	10.53	0.000
	After	0.929	0.936	−1.61	0.106
Agricultural production	Before	0.868	0.703	17.74	0.000
	After	0.869	0.869	−0.04	0.966
Family size	Before	4.359	4.050	6.06	0.000
	After	4.347	4.278	1.94	0.052
House value	Before	10.880	10.889	−0.13	0.894
	After	10.879	10.868	0.25	0.802
Consumption	Before	10.247	10.322	−3.18	0.001
	After	10.244	10.233	0.66	0.510

**Table 5:** PSM analysis of the effects of natural disasters on middle-aged and older adults’ mental health.

Variable	Kernel Matching	Local Linear Matching	Radius Matching	Nearest Neighbor Matching (k = 1)	Nearest Neighbor Matching (k = 4)
Disaster_d	−0.422 ***	−0.356 **	−0.408 ***	−0.477 ***	−0.416 ***
	(0.134)	(0.170)	(0.137)	(0.170)	(0.142)

Note: *** *p* < 0.01, ** *p* < 0.05.

**Table 6:** Robustness test results.

Variable	Dependent Variable: Mental Health		Dependent Variable: Mental Health (Dummy)			
	OLS	OLS	Probit	Probit	Probit	Probit
Disaster_d	−0.058 ***		−0.143 ***		−0.054 ***	
	(0.017)		(0.033)		(0.012)	
Disaster_n		−0.042 ***		−0.086 ***		−0.032 ***
		(0.005)		(0.009)		(0.035)
Control variable	YES	YES	YES	YES	YES	YES
Constant	−0.371 ***	−0.359 ***	−0.545 **	−0.540 **		
	(0.126)	(0.125)	(0.223)	(0.223)		
Observations	8721	8721	8721	8721	8721	8721
Adjusted R2	0.044	0.050				

Note: *** *p* < 0.01, ** *p* < 0.05. OLS represents ordinary least squares.

**Table 7:** Heterogeneous effects of natural disaster by education level.

Variable	Education		Education	
	Low	High	Low	High
Disaster_d	−0.423 *	−0.294 **		
	(0.218)	(0.146)		
Disaster_p			−0.318 ***	−0.225 ***
			(0.059)	(0.045)
Control variable	YES	YES	YES	YES
Constant	−5.270 ***	−1.052	−5.102 ***	−1.047
	(1.456)	(1.126)	(1.448)	(1.123)
Observations	3720	5001	3720	5001
Adjusted R2	0.021	0.027	0.028	0.032

Note: *** *p* < 0.01, ** *p* < 0.05, * *p* < 0.10.

**Table 8:** Heterogeneous effects of natural disaster by agricultural production status.

Variable	Agricultural Production		Agricultural Production	
	YES	NO	YES	NO
Disaster_d	−0.408 ***	−0.039		
	(0.140)	(0.269)		
Disaster_n			−0.288 ***	−0.097
			(0.039)	(0.095)
Control variable	YES	YES	YES	YES
Constant	−2.656 ***	−7.892 ***	−2.575 **	−7.700 ***
	(1.007)	(1.903)	(1.002)	(1.912)
Observations	7221	1500	7221	1500
Adjusted R2	0.042	0.052	0.048	0.053

Note: *** *p* < 0.01, ** *p* < 0.05.

**Table 9:** Natural disasters and happiness.

Variable	Dependent Variable: Life Satisfaction			
	OLS	OLS	Ordered Probit	Ordered Probit
Disaster_d	−0.202 ***		−0.097 ***	
	(0.059)		(0.027)	
Disaster_n		−0.144 ***		−0.066 ***
		(0.017)		(0.008)
Control variables	YES	YES	YES	YES
Constant	3.685 ***	3.724 ***		
	(0.417)	(0.414)		
Observations	8721	8721	8721	8721

Note: *** *p* < 0.01. OLS represents ordinary least squares.

**Table 10:** Natural disasters and life satisfaction.

Variable	Dependent Variable: Life Satisfaction			
	(1)	(2)	(3)	(4)
	OLS	OLS	Order Probit	Order Probit
Disaster_d	−0.112 ***		−0.121 ***	
	(0.026)		(0.028)	
Disaster_n		−0.060 ***		−0.064 ***
		(0.008)		(0.008)
Control variables	YES	YES	YES	YES
Constant	2.190 ***	2.190 ***		
	(0.186)	(0.185)		
Observations	8721	8721	8721	8721

Note: *** *p* < 0.01. OLS represents ordinary least squares.

## Data Availability

Data used in this paper can be found from the China Family Panel Survey, http://www.isss.pku.edu.cn/cfps/ (accessed on 30 December 2021).
